# Supplementary material for: A novel algorithm to detect non-wear time from raw accelerometer data using deep convolutional neural networks
Source: Sci Rep. 2021 Apr 23;11:8832. doi: 10.1038/s41598-021-87757-z (PMC8065130; doi:10.1038/s41598-021-87757-z)
Supplement: Supplementary file 1 — Supplementary Information. [file 41598_2021_87757_MOESM1_ESM.pdf]

Supplementary Information

A novel algorithm to detect non-wear time from raw accelerometer data using deep convolutional neural networks

Shaheen Syed[1,*], Bente Morseth[2], Laila A. Hopstock[3], and Alexander Horsch[1]

[1]Department of Computer Science, UiT The Arctic University of Norway, Tromsø, Norway
[2]School of Sport Sciences, Faculty of Health Sciences, UiT The Arctic University of Norway, Tromsø, Norway
[3]Department of Community Medicine, Faculty of Health Sciences, UiT The Arctic University of Norway, Tromsø, Norway
[*]corresponding author: shaheen.syed@uit.no

December 21, 2020

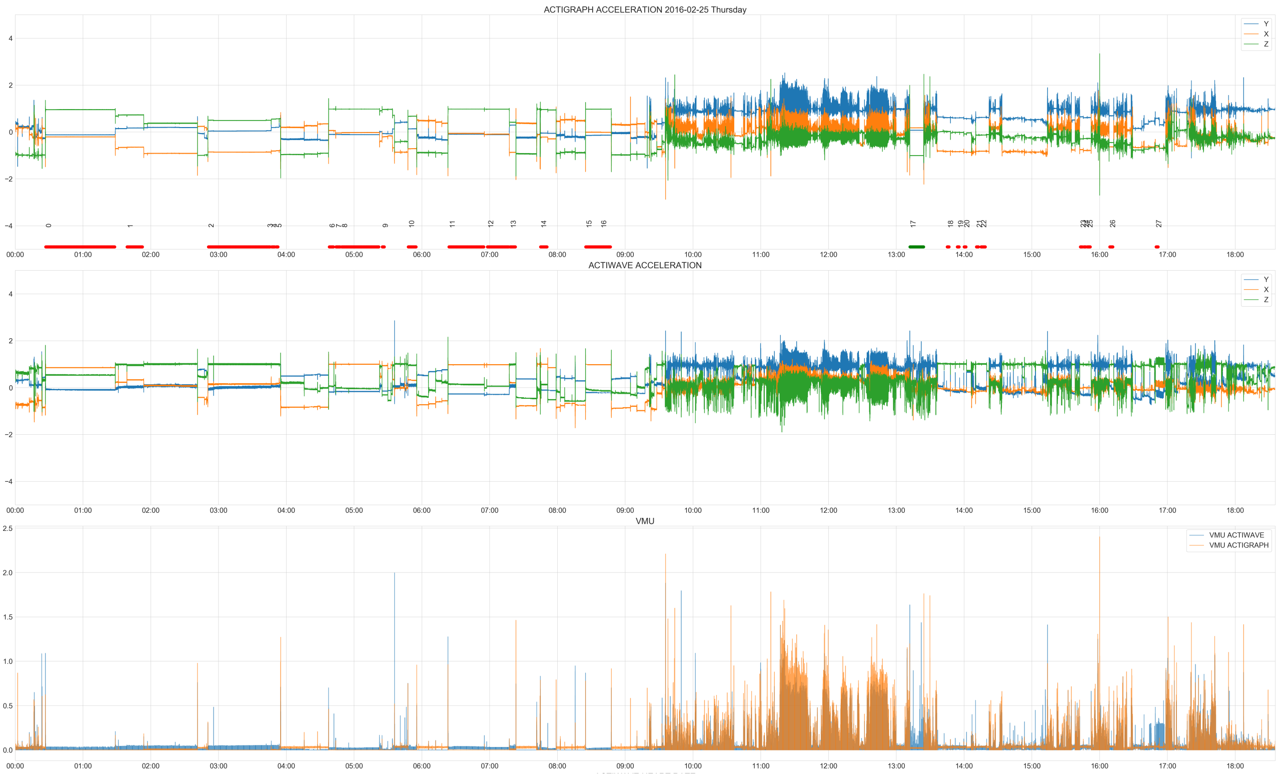

Figure S1: Overview of ActiGraph and Actiwave Cardio data for a single participant. The candidate non-wear episodes are shown in the top figure and indicated by a red or green horizontal line and numbered. The candidate non-wear episodes are duration of activity not exceeding a standard deviation threshold of 4.0 mg (0.004 g). A major challenge is to infer what happened during those episodes, and here the second accelerometer (Actiwave Cardio) provides additional information. By focusing on the discrepancies between the two signals, made more clear in the bottom graph that shows the vector magnitude (VMU), we know that candidate non-wear episode 17 (colored green) is true non-wear time since there is recorded activity measured by the Actiwave Cardio accelerometer.

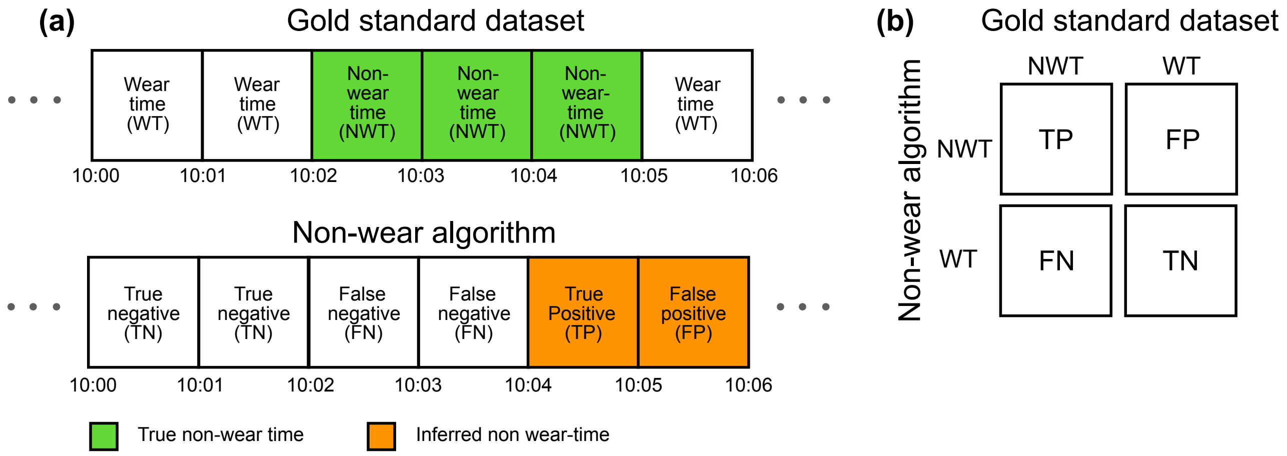

Figure S2: Graphical representation showing the correct and incorrect classifications of wear and non-wear time: **(a)** six minutes of labeled data is compared against six minutes of inferred data derived from a non-wear algorithm. Depending on the difference between the true labels and the inferred labels, each 1-minute interval is classified as a true negative or true positive if the labels match. Alternatively, intervals are classified as false negatives or false positives of the labels mismatch. **(b)** similarly as (a) but now shown as a confusion matrix.

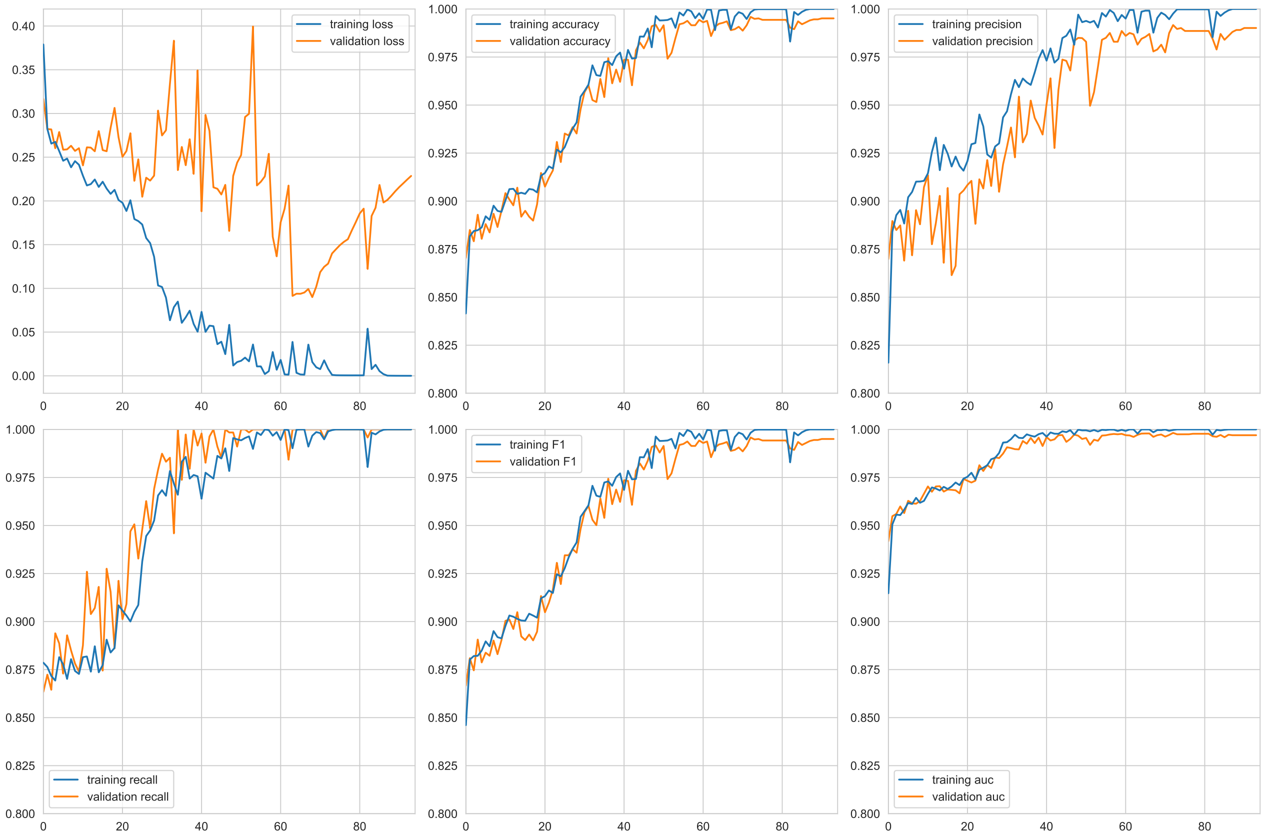

Figure S3: Overview of training (blue) and validation (orange) loss, accuracy, precision, recall, F1, and area under the curve (AUC) while training the CNN V2 model with a window length of 3 seconds. This model was set to train for 250 epochs with early stopping enabled when the validation loss did not increase for 10 epochs. The model was trained for a total of 94 epochs. In addition, the weights of the model with the best validation loss were restored. More information on early stopping can be found in the TensorFlow documentation at `https://www.tensorflow.org/api_docs/python/tf/keras/callbacks/EarlyStopping`

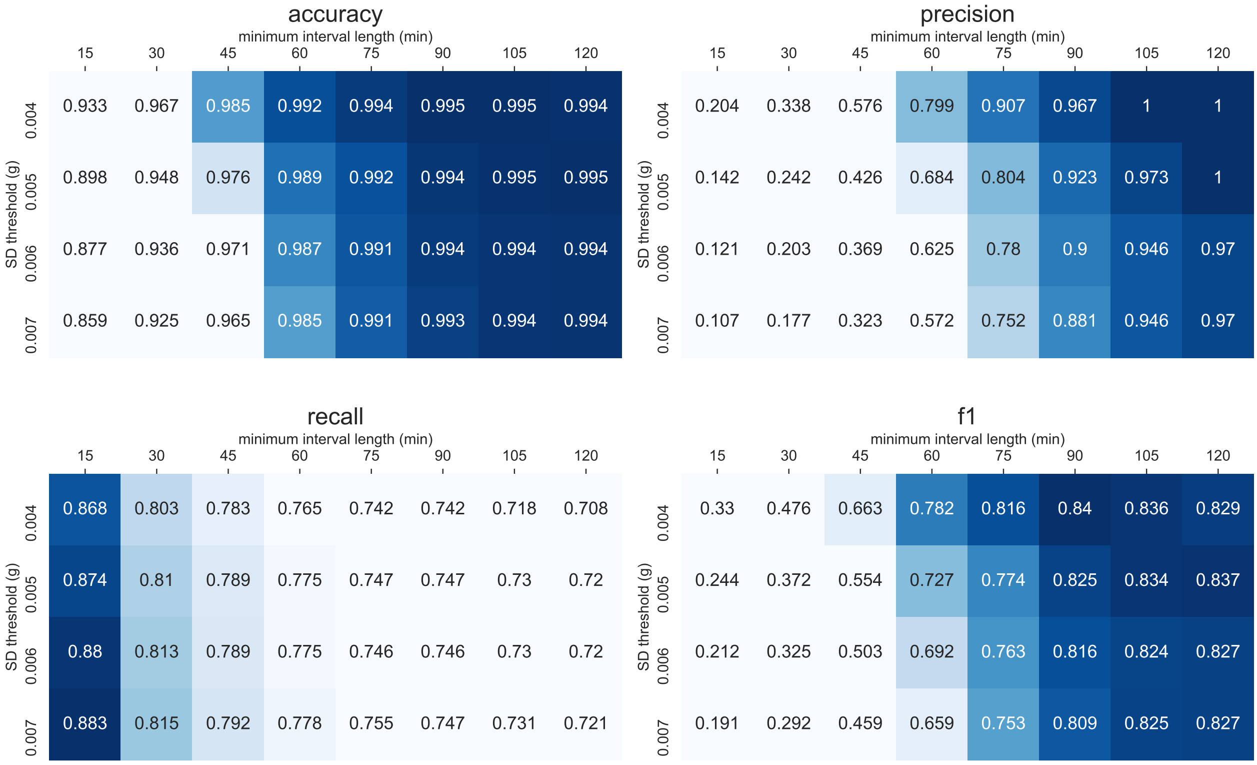

Figure S4: Classification performance metrics accuracy, precision, recall, and F1 for baseline models to detect non-wear time by using raw acceleration values in gravity units from all three orthogonal axes. This baseline model is referred to as the the XYZ non-wear algorithm.

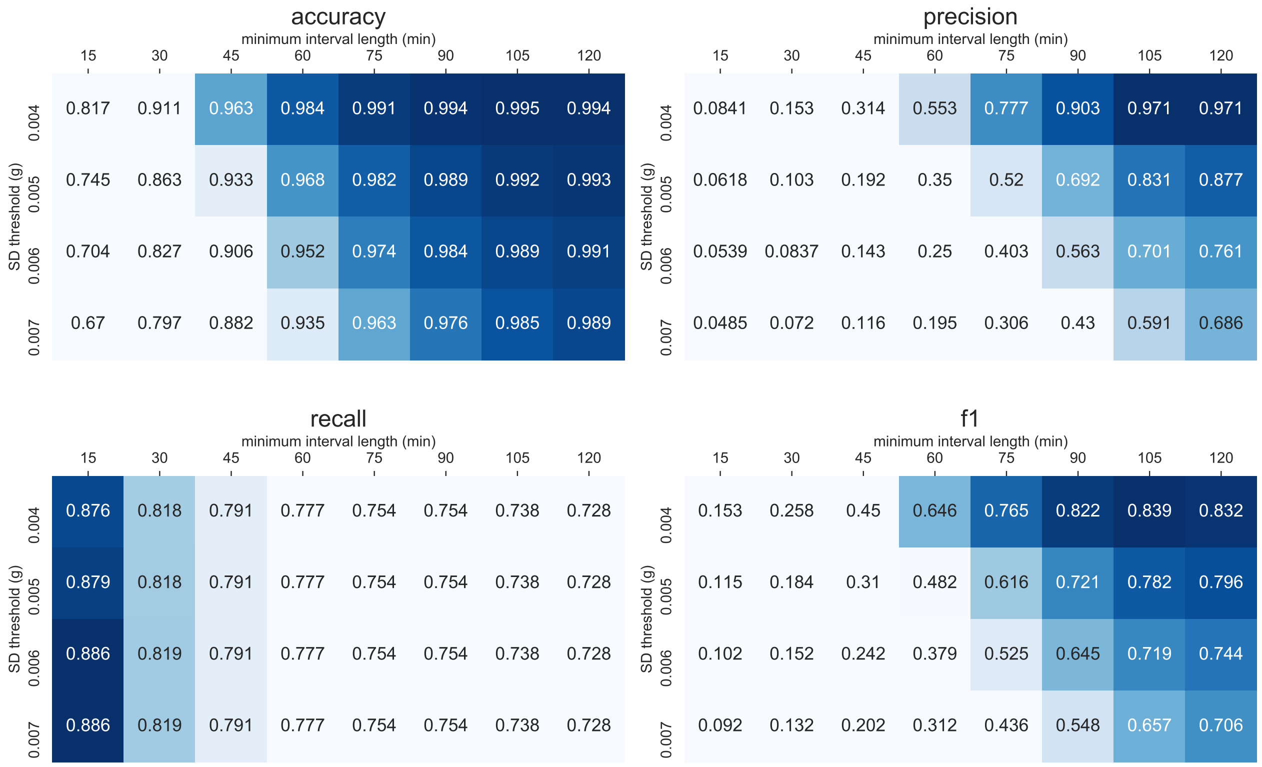

Figure S5: Classification performance metrics accuracy, precision, recall, and F1 for baseline models to detect non-wear time by using the vector magnitude units (VMU) calculated as $\sqrt{acc_x^2 + acc_y^2 + acc_z^2}$, where acc_x, acc_y, and acc_z refer to each of the orthogonal axes. This baseline model is referred to as the VMU non-wear algorithm.
